# The effects of acute garlic supplementation on the fibrinolytic and vasoreactive response to exercise

**DOI:** 10.1186/s12970-015-0084-9

**Published:** 2015-05-14

**Authors:** C. J. Womack, D. J. Lawton, L. Redmond, M. K. Todd, T. A. Hargens

**Affiliations:** Human Performance Laboratory, Department of Kinesiology, James Madison University, 261 Bluestone Dr, MSC 2302, Harrisonburg, VA 22807 USA

## Abstract

**Background:**

The purpose of this project was to examine the effects of acute garlic supplementation on fibrinolysis and vasoreactivity both at rest and following maximal exercise.

**Methods:**

Eighteen healthy trained males (20.9 ± 2.2 years, 178 ± 7.7 cm, 75.5 ± 9.6 kg, VO_2_max = 59.8 ± 6.7 ml • kg^−1^ • min^−1^) performed a graded treadmill test to volitional exhaustion. Blood samples were taken at rest, within two minutes post-exercise, and one hour post-exercise. Eleven of the subjects also had a brachial vasoreactivity test performed immediately after the blood sample to assess flow-mediated dilation (FMD) of the brachial artery. Participants were randomly assigned to ingest either 900 mg of powdered garlic or a placebo three hours before the exercise session. The supplement was distributed in a double-blind, crossover fashion. Participants repeated the protocol with the other treatment after a 14-day washout period. Paired t-tests were used to compare VO_2_max between the two trials. A two-factor (treatment and time) repeated measures analysis of variance (ANOVA) was used to assess changes in FMD, tPA activity, tPA antigen, and PAI-1 activity. A priori statistical significance was set at P <0.05.

**Results:**

VO_2_max was greater for the garlic treatment trial vs. placebo (Placebo = 59.8 ± 6.7 ml • kg^−1^ • min^−1^; Garlic = 61.4 ± 6.6 ml • kg^−1^ • min^−1^). There was no main effect for treatment and no treatment x time interaction for FMD or any fibrinolytic variables examined.

**Conclusion:**

Acute garlic supplementation does not alter vasoreactivity, fibrinolytic potential or the fibrinolytic response to exercise in young healthy trained males. Acute garlic supplementation does, however, cause a small but statistically significant increase in VO_2_max. It remains unclear if this increase in VO_2_max is of functional importance.

## Introduction

Prior epidemiological studies have observed that garlic improves the risk for the development of cardiovascular disease (CVD) [1, 2]. This positive health outcome is likely due to many physiological effects. Specifically, garlic can positively affect plasma lipids [3-5], cause reductions in blood pressure [5-8], and affect changes in hemostasis [9-16]. Furthermore, some of the blood pressure and hemostatic effects are evident acutely, after a single dose [7, 9, 12, 13, 16].

The relative risk of myocardial infarction is 5.9 fold greater in the hour after vigorous exercise [17]. The purported causes of these exertion-related ischemic events are thrombosis, coronary vasospasm, or both [18]. Thus, factors that impact the vascular and/or hemostatic responses to exercise are clinically significant. Fibrinolysis is the capacity to lyse inappropriate or excessive clotting. The degree of fibrinolytic potential is influenced in humans by tissue plasminogen activator (tPA) which converts plasminogen to plasmin and plasminogen activator inhibitor 1 (PAI-1), which is the main circulating inhibitor of tPA. Early studies on garlic supplementation reported substantial increases in fibrinolytic activity after acute and chronic supplementation [9-12, 14, 15]. Although the results of these studies are illuminating, the changes in fibrinolytic activity were assessed globally, without specific assays for tPA or PAI-1. Thus, the effects of garlic supplementation on the specific components of the fibrinolytic system are not known. Furthermore, it is not known whether garlic affects the fibrinolytic response to exercise.

There is evidence that garlic elicits vascular benefits through the enhanced availability of nitric oxide (NO). Garlic’s influence on NO synthase (eNOS) has been proposed as a potential mechanism for this enhanced availability, and a single dose may be enough to elicit this response [19, 20]. Das et al [21] found that the active compounds in garlic can increase eNOS activity intracellularly, subsequently increasing NO production. Similar findings by Morihara et al [20] showed that aged garlic extract (AGE) significantly increased NO levels in the plasma when compared to baseline values. Williams et al [22] observed that two weeks of garlic supplementation significantly increased flow-mediated dilation in men with coronary artery disease. However, the effects of acute garlic supplementation on this measure of vasoreactivity and its effect on the vasoreactive response to exercise are unknown. The purpose of the present study was to evaluate the acute effects of garlic supplementation on tPA, PAI-1, and flow-mediated vasodilation, both at rest and in response to exercise. It was hypothesized that the garlic supplementation would increase fibrinolysis and flow-mediated dilation.

## Methodology

### Participants

Eighteen healthy males (age = 20.9 ± 2.2; height = 178.0 ± 7.7 cm; weight = 75.5 ± 9.6 kg) from the James Madison University community and the surrounding Harrisonburg area completed this study. The original cohort consisted of 20 subjects but two of these subjects dropped out. All participants were trained in that they either: A) participated in three or more days of vigorous activity for at least twenty minutes per day or B) participated in seven or more days of any combination of moderate-intensity or vigorous-intensity activities achieving a minimum total physical activity of 3000 MET-min • wk^−1^. Exclusion criteria included: any known cardiovascular, pulmonary, or metabolic disease; current tobacco use; current use of any medication known to influence fibrinolysis; infection; fever, or illness within two weeks prior to testing; garlic or flour allergy; and any other medical condition that could compromise safety. Participants were provided with written and verbal information about the experimental procedures, including potential risks, prior to completing an informed consent form. All procedures were approved by the James Madison University Committee on Research Involving Human Subjects prior to testing.

### Supplementation

Participants were given a packet containing either the placebo treatment (PT), which consisted of three gel capsules containing flour, or the garlic treatment (GT), which consisted of three 300 mg capsules of Kyolic Brand powdered aged garlic extract (Mission Viejo, CA). These treatment packets were administered in a double-blind, cross-over fashion with the order structured to ensure that an equal number of participants performed the placebo treatment and the garlic treatment first. Participants were instructed to ingest all of the tablets with a glass of water three hours prior to testing. All tests were performed before 10 AM to minimize the effect of diurnal variations on fibrinolysis [23] and each participant was tested at the same time of day for both trials.

### Dietary and exercise controls

Throughout the duration of the study participants were instructed to: 1) choose a standard “self-selected” meal to be repeated for the twenty-four hours preceding the two treatment trials, 2) consume the last meal of the day no later than twelve hours prior to the treatment trials, 3) avoid consumption of alcohol twenty-four hours prior to each treatment trial, and 4) avoid exercise immediately prior to each treatment trial. For the self-selected meal, all participants completed a 24-hour dietary recall before the first and second trial. In twelve of the eighteen participants, at least 75 % of the food items from the day before the first trial were repeated on the day before the second trial. For the six participants who did not meet this criterion, there was no reported garlic ingestion the day prior to either trial. Each participant confirmed verbally that they did not exercise before the treatment trials. Participants were allowed to drink water *ad libitum* throughout the duration of the study.

### Exercise tests

Upon arrival at the laboratory, participants assumed a semi-recumbent position for 20 min prior to obtaining a baseline blood sample and measuring flow-mediated dilation (FMD) of the brachial artery. Participants then completed a graded exercise test (GXT) to volitional fatigue on a treadmill. Blood sampling and FMD measurements were then obtained immediately after exercise and again 1 hour after exercise cessation. Participants repeated the protocol with the other treatment two weeks later.

The GXT was performed on a Stairmaster Clubtrack 612 treadmill (Kirkland, WA). The test began at an initial speed of 67 m • min^−1^. Speed was then increased at a rate of 13.4 m • min^−1^ each minute until 161 m • min^−1^ was reached. At this point, the treadmill speed remained constant while the elevation increased at a rate of 3.0 % • min^−1^ until 15.0 % grade was reached. Speed was then increased once again at a rate of 13 m • min^−1^ until volitional exhaustion. VO_2_ was continuously monitored with a Sensormedics Spectra metabolic cart (Yorba Linda, CA) and the highest one-minute average was used to determine VO_2_max. This protocol has been previously shown to elicit profound fibrinolytic responses [24, 25].

### Blood sampling

For each blood sample, 5 ml of venous blood was collected in a tube treated with acidified citrate (Biopool International; Ventura, Calif) and an additional 5 ml of blood was collected in an ethylenediaminetetraacetic acid (EDTA) tube. Whole blood collected in EDTA was analyzed for hematocrit using the microhematocrit method. The remainder of the EDTA sample and the blood collected in an acidified citrate tube were spun in a refrigerated centrifuge (Fisher Scientific accuSpin 3r) operating at 4°C and 10,000 rpm for 20 min to obtain platelet-poor plasma. Plasma was alliquoted and stored at -80°C until assayed. Fibrinolytic potential was determined by assaying for tPA activity, tPA antigen, and PAI-1 activity. tPA activity was determined by using an amidolytic activity assay (Biopool International; Ventura, Calif). tPA antigen and PAI-1 activity were determined using an enzyme-linked immunosorbent assay (ELISA) (American Diagnostica, Greenwich, Conn and Diapharma respectively). All values for tPA and PAI-1 were corrected for plasma volume changes [26].

### Flow mediated dilation

Flow mediated dilation of the brachial artery was measured on three separate occasions during each trial: at resting prior to the exercise test, beginning within 2 minutes post exercise test, and 1 hour post exercise test. PerformancePlus ECG Diagnostic Electrodes (Vermed: Bellows Falls, VT) for an electrocardiograph (ECG) were placed on each participant at the left arm, right arm, and left hip for the purposes of coordinating blood flow with cardiac cycles. The distance between the right olecranon process and the elbow was measured, and a DC-6 Diagnostic Ultrasound System (ShenZhen Mindray Bio-Medical Electronics Co., Ltd.: ShenZhen, China) probe was placed at approximately 1/3 of this distance from the elbow to locate the brachial artery. Once the brachial artery was located and an adequate image achieved, the ultrasound probe’s position was marked lightly on the participant’s skin using a permanent marker to allow for quick relocation of the artery during the trial and standardization of the measurements. For each FMD measurement, a DS400 Aneroid Sphygmomanometer (Hokanson: Bellevue, WA) was placed on the forearm, distal to the right brachial artery [27]. The cuff was inflated to 200 - 250 mmHg (≥25 to 50 mmHg above systolic arterial pressure) and kept inflated for a period of five minutes [28]. Blood flow measurements were initiated at least ten seconds prior to cuff release and post occlusion data collection was continued for ≥ two minutes [29]. The cuff was removed after completion of each FMD measurement during the trial.

All data was collected with the use of Microsoft AM Capture software (Version 8 downloaded from Pennacle Systems Division of Avid Technologies, Inc. New York) at a rate of 8 frames per second. Microsoft AM Capture files were converted using Movavi Video Converter software (Version 10 downloaded from Movavi, Novosibirsk Province, Russian Federation) and analyzed using Brachial Analyzer for Research software (Medical Imaging Applications, LLC. Coralville, IA).

Baseline vessel diameters were recorded as the mean diameter recorded during the ten seconds prior to cuff release. This method for measuring baseline diameter values has been successfully used elsewhere [27, 30-34]. Post-occlusion diameter measurements were recorded and averaged at four-second intervals (32 frames) during the first twenty seconds post-occlusion and at five-second intervals (40 frames) for the remainder of the two minutes. Peak diameter was recorded as the highest average diameter measured from among the four- or five-second intervals during the two minutes of measurement following cuff release [29]. Time to peak diameter was estimated as the midpoint of the interval in which peak diameter was recorded. This method for measuring vessel diameters and computing FMD has been shown to be equally valid and reliable as QRS-gating brachial artery diameter measurements [35]. Diameter measurements analyzed with the Brachial Analyzer for Research were used to calculate FMD, which was calculated as [(peak diameter – baseline diameter)/baseline diameter] and recorded as a percent of change.

### Statistical analysis

The primary dependent variables studied for the present investigation were tPA activity, tPA antigen, PAI-1 activity, and FMD. A two-factor (treatment and time) repeated measures analysis of variance (ANOVA) was used to assess changes in these variables. Paired t-tests were used to compare our secondary outcome variable (VO_2_max) between the two trials. A priori statistical significance was set at P < 0.05. All statistical analysis was performed using IBM SPSS statistical software.

## Results

**Fig. 1:**
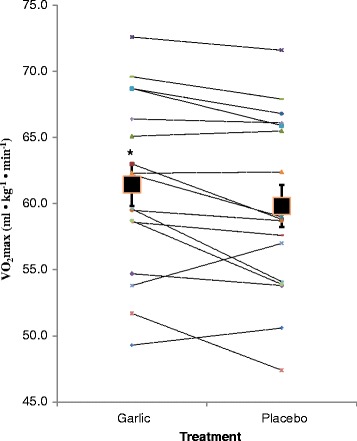
Mean values for VO_2_max for the garlic and placebo conditions. The individual responses are also displayed as dashed lines. *- Significantly higher than placebo (P < 0.05)

Gas exchange data was available for seventeen of the subjects. Fig. 1 displays average relative VO_2_max for both conditions, along with the individual data. VO_2_max was significantly (p < 0.05) greater with GT [PT = 59.8 ± 6.7 ml • kg^−1^ • min^−1^, GT = 61.4 ± 6.6 ml • kg^−1^ • min^−1^].

### Fibrinolytic potential

One participant was a consistent outlier (greater than two standard deviations above the mean) for tPA antigen and PAI-1 activity and was excluded from the analysis of fibrinolytic variables. A Shapiro-Wilk test demonstrated that the data was not normally distributed for tPA antigen or PAI-1 activity for at least one time point. These data were log-transformed prior to ANOVA. Further analysis revealed that PAI-1 activity was still abnormally distributed after the log-transformation. The next highest outlier was removed from the analysis of fibrinolytic variables and normalcy was then achieved for PAI-1.

**Fig. 2:**
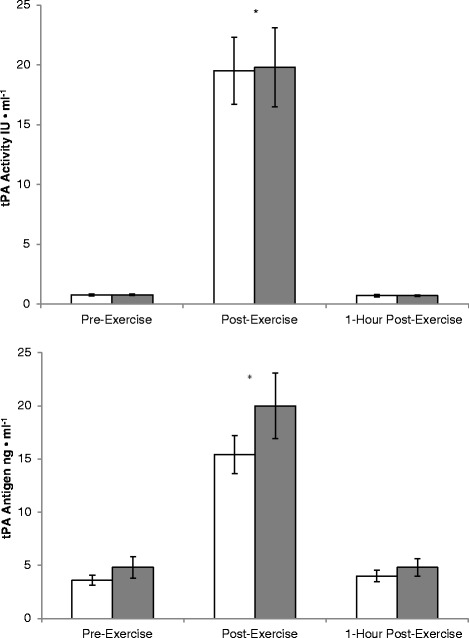
Mean plasma levels of tPA Activity (IU • ml^−1^) and tPA antigen (ng • ml^−1^) pre-exercise, post-exercise, and 1-hour post-exercise for the placebo (white bars) and garlic (gray bars) conditions. *- Significantly higher than pre-exercise (P < 0.05)

Figure 2 displays the mean tPA activity and tPA antigen response for the PT and GT. A significant (p < 0.05) main effect was observed for time as tPA activity increased from pre-exercise [PT = 0.74 ± 0.08 IU • ml^−1^, GT = 0.76 ± 0.07 IU • ml^−1^] to post-exercise [PT = 19.5 ± 2.8 IU • ml^−1^, GT = 19.8 ± 3.3 IU • ml^−1^]. Compared to pre-exercise values, there was no elevation in tPA activity 1-h post-exercise [PT = 0.69 ± 0.10 IU • ml^−1^, GT = 0.69 ± 0.06 IU • ml^−1^]. tPA antigen significantly (p < 0.05) increased from pre-exercise [PT = 3.6 ± 0.46 ng • ml^−1^, GT = 4.8 ± 1.0 ng • ml^−1^] to post-exercise [PT = 15.4 ± 1.8 ng • ml^−1^, GT = 20.0 ± 3.1 ng • ml^−1^]. One hour post-exercise tPA antigen was reduced to baseline as it was not significantly different than pre-exercise values [PT = 4.0 ± 0.52 ng • ml^−1^, GT = 4.8 ± 0.80 ng • ml^−1^]. There was no main effect for treatment and no treatment x time interaction for these variables (p > 0.05).

**Fig. 3:**
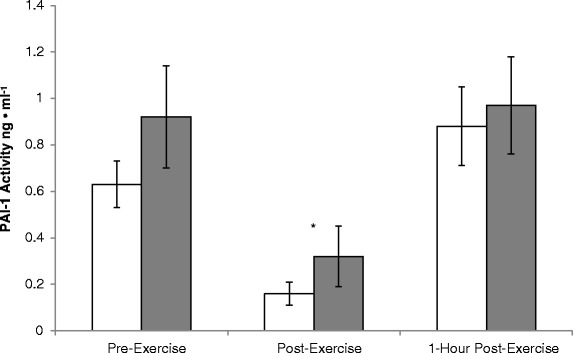
Mean plasma levels of PAI-1 Activity (ng • ml^−1^) pre-exercise, post-exercise, and 1-hour post-exercise for the placebo (white bars) and garlic (gray bars) conditions. *- Significantly lower than pre-exercise (P < 0.05)

Figure 3 displays the mean PAI-1 activity response for the PT and GT. A significant (p < 0.05) main effect was observed for time as PAI-1 activity significantly (p < 0.05) decreased from pre-exercise [PT = 0.63 ± 0.10 ng • ml^−1^, GT = 0.92 ± 0.22 ng • ml^−1^] to post-exercise [PT = 0.16 ± 0.05 ng • ml^−1^, GT = 0.32 ± 0.13 ng • ml^−1^]. There was no difference in pre-exercise PAI-1 activity and PAI-1 activity 1-h post-exercise [PT = 0.88 ± 0.17 ng • ml^−1^, GT = 0.97 ± 0.21 ng • ml^−1^]. There was no significant main effect (p > 0.05) for treatment or a significant (p > 0.05) treatment x time interaction for PAI-1 activity.

### Flow-mediation dilation

Seven of the eighteen participants were excluded from the analysis of FMD-related variables. Six of the seven were unable to be analyzed due to poor image quality or missing data points. A Shapiro-Wilk test revealed that the remaining 12 data points were abnormally distributed. This was corrected by removing another participant that represented an extreme outlier (two data points were approximately three standard deviations from the mean). Removal of this participant then resulted in normally distributed data.

Average change in FMD and peak diameter are summarized in Table 1. No significant main effect for treatment (p > 0.05) or time (p > 0.05) or a treatment x time interaction were found (p > 0.05) for these variables.

**Table 1:** Mean FMD, peak diameter and time to peak diameter (± Std. Dev) for both conditions at all three time-points

Variable		Resting	Post-exercise	1-hour post-exercise
FMD (mm)	Garlic	0.068 ± 0.032	0.091 ± 0.119	0.084 ± 0.097
	Placebo	0.071 ± 0.051	0.071 ± 0.084	0.108 ± 0.097
Peak diameter (mm)	Garlic	4.241 ± 0.388	4.307 ± 0.383	4.293 ± 0.443
	Placebo	4.337 ± 0.516	4.322 ± 0.348	4.463 ± 0.285
Time to peak diameter (sec)	Garlic	56.36 ± 19.24	66.36 ± 13.43	60.00 ± 14.66
	Placebo	51.82 ± 16.77	69.09 ± 12.81	61.36 ± 13.05

Time to peak diameter data is also summarized in Table 1. No significant main effects for treatment (p > 0.05) or treatment by time interactions were found (p > 0.05). A significant main effect for time (p < 0.05) was observed as the time to peak diameter was longer immediately after exercise.

## Discussion

The primary finding of this study is that acute garlic supplementation does not improve resting fibrinolytic potential or vasoreactivity; nor does it affect the fibrinolytic or vasoreactive response to exercise in young, healthy males. Similarly, Legnani and co-workers [16] did not observe improvements in tPA activity with acute garlic supplementation. In that investigation, the participant pool was young and healthy and supplemented with 900 mg of a garlic powder preparation four hours prior to evaluation. In contrast, several studies have observed enhancement of the fibrinolytic system within a few hours after the consumption of garlic. Euglobulin clot lysis time (ECLT) has been observed to decrease following acute administration of garlic oil [9] or raw garlic [13]. However, in one of these studies, the improved ECLT from garlic occurred following butter ingestion [9], while the other study was performed on participants with heart disease [13]. Thus, the discrepancy of results could be explained by methodology and population.

It is also possible that the acute garlic supplementation regimen in the present study could have possibly affected another hemostatic factor, such as fibrinogen or urinary plasminogen activator, which would also affect ECLT but does not specifically affect tPA or PAI-1. However, Kiesewetter et al. [36] did observe significant (56 % and 51 %, respectively) increases in tPA activity five hours after acute supplementation of 300 mg and 600 mg of garlic in young, healthy males. Furthermore, Jung and coworkers [37] measured fibrinolytic potential following acute garlic supplementation and observed about an 86 % increase in tPA activity after five hours in a similar subject sample. It is possible, therefore, that acute garlic supplementation does not impact hemostasis until at least five hours after ingestion as data reported from our study and from Legnani et al were four and three hours after supplementation respectively.

In addition to the lack of hemostatic effect in the present study, acute garlic supplementation also failed to have an impact on brachial artery vasoreactivity. In prior studies, garlic improved synthesis of NO in platelets and tissue preparations [21] as well as production in mice [19, 20]. Furthermore, chronic AGE supplementation has been shown to improve brachial vasoreactivity in individuals with acute hyperhomocysteinemia. Therefore, prior research clearly suggests that garlic is capable of improving endothelial function and that this improvement can lead to increased vasoreactivity. However, the fact that the current study was performed on young, healthy individuals suggests that the effects of an acute dose in a healthy population are not sufficient to observe significant changes. Furthermore, it is possible that the garlic supplementation increased NO synthesis, but not to the extent that it improved brachial vasoreactivity in this population. Finally, it is possible that improvements would have been observed if a sample with cardiovascular disease, or another condition that compromises vasoreactivity, were studied but this possibility can only be addressed with future research.

In the present study, we observed a significant increase in the time to peak diameter immediately following exercise. Rognmo et al [38] observed a 38 % reduction from baseline in FMD values one-hour post-exercise in trained subjects. This occurred despite the fact that NO was increased 93 % at 1-hour post-exercise, along with large arterial diameters at this same time point. It was suggested that this large arterial diameter, combined with high levels of circulating NO in the plasma, may have dilated the vessel to maximal or near maximal diameter prior to FMD measurement. Thus it is possible that in this population of young, healthy males, the exercise increased arterial diameter and peak blood flow to a point that could not be further enhanced by acute garlic supplementation.

Evidence from Silber et al [39] may also explain why there was no significant difference (*p* = 0.524) in peak diameters across all time-points. The current study used young, healthy, trained males similar in characteristics to the trained athletes in the study by Rognmo et al [38]. These athletes had larger brachial diameters than the sedentary controls, and were less prone to increased dilation due to increased shear stress as their diameters were already large enough to accommodate increased blood flow to exercising muscles [38]. As the participants in the current study were of similar training status, it is probable that they too had relatively large brachial artery diameters. If larger arteries dilate to a lesser extent than smaller arteries [39], there would be less change in dilation from resting to peak dilation.

An unexpected secondary finding of the present study is that acute garlic supplementation causes a slight but significant increase in VO_2_max in young, healthy, trained males without significantly increasing their treadmill time. Garlic has been suggested as an anti-fatigue agent, although the specific mechanism of this effect has not been elucidated [40]. Of the 17 participants whose gas exchange data was analyzed, thirteen of them achieved a higher relative VO_2_max during the garlic trial than during the placebo trial. The average relative VO_2_max was 59.8 ml • kg^−1^ • min^−1^ and 61.4 ml • kg^−1^ • min^−1^ for the placebo and garlic trials, respectively. Ince & colleagues [41] administered a single dose of garlic to ten college-aged endurance athletes and observed a significant increase in VO_2_max (57.3 ml • kg^−1^ • min^−1^ for the garlic trial and 55.6 ml • kg^−1^ • min^−1^ for the placebo trial) five hours after garlic ingestion. It was speculated that the observed differences between the garlic and placebo trials were due to improvements in the fluidity of the blood whereby whole-blood viscosity was decreased due to garlic-induced reductions in fibrinogen concentration. However, fibrinogen was not measured by Ince & colleagues [41] or in the current study so this possibility cannot be proven. Interestingly, Morihara & coworkers [20] also examined the impact of aged garlic extract (AGE) on succinate dehydrogenase (SDH) activity. AGE significantly increased SDH activity by 40 % in the gastrocnemius muscle of the rats engaging in repeated endurance exercise on a mechanical treadmill 5 times per week for 4 weeks as compared to the control exercising rats which did not receive AGE. This elevation in SDH activity may indicate AGE enhances aerobic metabolism of skeletal muscle during exercise, which in turn may account for the increase in VO_2_max observed in the present study.

The major limitation of the present study is that the findings are specific to healthy young males with a high cardiorespiratory endurance. It is entirely possible that individuals with impaired fibrinolysis and/or diminished brachial vasoreactivity would experience improvements in one or both of these parameters with acute garlic supplementation. However, our findings do strongly suggest that acute garlic supplementation does not impact tPA, PAI-1 or brachial vasoreactivity in the studied population.

The functional importance of the observed improvements in VO_2_max is uncertain. The present study found a 1.6 ml • kg^−1^ • min^−1^ increase in VO_2_max without a corresponding improvement in treadmill time. Conversely, Ince & colleagues [41] observed a 2 ml • kg^−1^ • min^−1^ increase in VO_2_max as well as a 53.6 s increase in treadmill time. Both studies used groups of healthy trained college aged males. Future studies should seek to replicate the present findings to see if the lack of improved treadmill time persists.

## Conclusion

In summation, the present study does not support the hypothesis that acute supplementation with garlic enhances the resting of post-exercise fibrinolysis or vasoreactivity in young, healthy males. However it does appear that acute garlic supplementation does result in a small but significant increase in VO_2_max in this population. Future studies should evaluate whether these improvements in VO_2_max are of any functional importance.
